# Prognostic Impact of PD-1 and Tim-3 Expression in Tumor Tissue in Stage I-III Colorectal Cancer

**DOI:** 10.1155/2020/5294043

**Published:** 2020-05-14

**Authors:** Wentao Kuai, Xinjian Xu, Jing Yan, Wujie Zhao, Yaxing Li, Bin Wang, Na Yuan, Zhongxin Li, Yitao Jia

**Affiliations:** ^1^Third Department of Oncology, Hebei General Hospital, 050051 Shijiazhuang, China; ^2^Hebei North University, 075000 Zhangjiakou, China; ^3^Department of Thoracic Surgery, The Fourth Hospital of Hebei Medical University, 050035 Shijiazhuang, China; ^4^The First Affiliated Hospital of Hebei Northern University, 075000 Zhangjiakou, China; ^5^Second Department of Surgery, The Fourth Hospital of Hebei Medical University, 050011 Shijiazhuang, China

## Abstract

**Background:**

Programmed cell death receptor 1 (PD-1) and T cell immunoglobulin mucin-3 (Tim-3) are considered as important immunosuppressive molecules and play an important role in tumor immune escape and cancer progression. However, it remains unclear whether PD-1 and Tim-3 are coexpressed in stage I-III colorectal cancer (CRC) and how they impact on the prognosis of the disease.

**Materials and Methods:**

A total of two cohorts with 451 patients who underwent surgery for stage I-III CRC treatment were enrolled in the study. Among which, 378 cases were from The Cancer Genome Atlas (TCGA) database and 73 cases were from the Fourth Hospital of Hebei Medical University (FHHMU) cohort. The mRNA expressions of PD-1 and Tim-3 in tumor tissue in stage I-III CRC were obtained from TCGA database. Immunohistochemistry was used to assess the expressions of PD-1 and Tim-3 in tumor tissue in stage I-III CRC in the FHHMU cohort. Interactive relationships between PD-1 and Tim-3 were retrieved through the online STRING database, which was used to study the interactions between proteins. DAVID, consisting of comprehensive biological function annotation information, was applied for the GO and KEGG pathway enrichment analysis of the interactive genes.

**Results:**

In the FHHMU cohort, the high expressions of PD-1 and Tim-3 were, respectively, found in 42.47% and 84.93% of stage I-III CRC tissue. PD-1 was significantly associated with age, primary site, and lymphatic metastasis. Tim-3 was closely related to the primary site. Correlation analysis showed that PD-1 and Tim-3 were positively correlated (*r* = 0.5682, *P* < 0.001). In TCGA cohort, PD-1 and Tim-3 were associated with the prognosis of CRC patients in terms of 5-year survival (*P* < 0.05). In the FHHMU cohort, the 5-year survival of patients with high levels of PD-1 and Tim-3 was 54.84% and 65.85%, respectively. Among which, the high expression of PD-1 was associated with poor prognosis (5-year OS: 54.84% vs. 88.10%, *P* = 0.003). The 5-year survival rate of CRC patients with coexpression of PD-1 and Tim-3 was 45.00%, which was significantly worse than non-coexpression (72.73%, 85.71%, and 90.48% separately). The functional network of PD-1 and Tim-3 primarily participates in the regulation of immune cell activation and proliferation, immune cell receptor complex, cell adhesion molecules, and T cell receptor signaling pathway.

**Conclusion:**

In summary, upregulation of PD-1 and Tim-3 in stage I-III CRC tumor tissue could be associated with the poor prognosis of patients. Those patients with coexpression of PD-1 and Tim-3 may have a significantly worse prognosis.

## 1. Introduction

Colorectal cancer (CRC) is the third most common malignancy and leads to more than 600,000 people deaths each year worldwide [1]. The prognosis of patients with operable CRC has improved significantly in the development of treatments such as surgery, chemotherapy, radiotherapy, and targeted therapy. However, greater than 40% of CRC patients develop local recurrence and distant metastasis after surgical treatment [2]. The prognosis of operable CRC patients is mainly related to postoperative tumor recurrence and distant metastasis. The main cause of tumor recurrence and distant metastasis is closely connected with the local immune status and malignant degree of tumor [3]. Mlecnik et al. found that the immunoscore could be considered as a predictor of response to chemotherapy in stage II and III CRC [4]. However, the prognosis of patients with CRC remains an urgent issue.

Recently, programmed cell death receptor 1 (PD-1) and T cell immunoglobulin mucin-3 (Tim-3) are considered as important immunosuppressive molecules. They play an important role in tumor immune escape and cancer progression and affect the prognosis of a variety of tumor patients [5, 6]. PD-1, a member of the B7/CD28 family, can be expressed in activated CD4^+^ T cells, CD8^+^ T cells, B cells, and NK T cells [5, 7]. Tumor cells (TCs) and their related stromal cells can express its ligands (PD-L1 or PD-L2). The combination of PD-1 and PD-L1/PD-L2 can inhibit the activation of lymphocytes and the production of cytokines, leading to the deletion of tumor-infiltrating cells (TILs) and induction of immunological tolerance [8, 9]. TILs are widely considered as a reflection of primary host immune response against solid tumors. However, the ligand-receptor interaction can inhibit activity of PD-1^+^ TILs and silence the immune system [10]. Yassin et al. have found that PD-1 is upregulated following tumor development and the increase of PD-1 expression is associated with tumor progression in inflammation-induced CRC in mice [11]. In addition, evidence has shown that high expression of PD-1 is associated with poor prognosis in primary central nervous system lymphoma (PCNSL) and esophageal cancer [12, 13]. It is revealed that PD-L1 could serve as the significant biomarker for poor prognosis and the adverse clinic-pathological features of CRC [14].

Tim-3, a member of the Tim family, was firstly discovered on the surface of Th1 cells and Tc1 cells [15]. Some scholars discovered that Tim-3 was also expressed in malignant tumor cells, such as kidney cancer and CRC [16, 17]. Tim-3 on the surface of immune cells binds to galectin-9, which promotes the apoptosis of Tim-3^+^ Th1 cells and induces the immune escape of tumor cells [18]. Growing evidence has shown that Tim-3 expressed in TCs plays an important role in tumor biology. For example, Tim-3 can directly promote the proliferation and invasion of CRC cells [19]. Knockdown of Tim-3 significantly reduces the cell proliferative rate of HCT116 and HT-29 cells [20]. Moreover, Shan and his colleague found that the expression intensity of Tim-3 in esophageal cancer was negatively correlated with the prognosis of patients [21]. It is found that Tim-3 is a critical mediator in the progression of CRC and could be the potential independent prognostic factor for CRC patients [10, 20].

However, it remains unclear whether PD-1 and Tim-3 are coexpressed and how they impact on the prognosis of CRC. In this study, we enrolled 378 cases in The Cancer Genome Atlas (TCGA) database and a cohort including 73 well-documented, clinically annotated CRC tumor specimens to investigate the expression of PD-1 and Tim-3 and explore the relationship between PD-1 and Tim-3 and the prognostic value of PD-1 and Tim-3 expressions in stage I-III CRC.

## 2. Materials and Methods

### 2.1. Patients and Samples

For TCGA cohort, the mRNA expressions of PD-1 and Tim-3 in CRC tumor tissue and clinical data of TCGA database were obtained from the website of the Cancer Genomics Browser of University of California Santa Cruz (https://genome-cancer.ucsc.edu/). Detailed PD-1 and Tim-3 expression data in 378 primary CRC tumors from patients were chosen from the updated TCGA database. Only those patients with fully characterized tumors, overall survival (OS), complete RNAseq information, without organ metastases, and undergoing radical surgery were included. The age, gender, tumor location, TNM stage, historical type, and OS were collected as clinic-pathological characteristics.

The Fourth Hospital of Hebei Medical University (FHHMU) cohort consists of 73 patients with histological-confirmed primary CRC who had undergone radical surgery. Only those patients with operable single tumors and without any evidence of metastasis at the time of diagnosis were enrolled. All patients were treated at FHHMU from January 2008 to February 2012. The follow-up time was from December 2017 and lasts for more than 5 years until patient death. The total survival time was from the date of diagnosis to the last follow-up or from the date of diagnosis to death or loss to follow-up. A total of 90 patients were monitored. Among which, 17 patients were lost to follow-up. Survival data of the remaining 73 patients was obtained. The follow-up rate was 81.1%, and the 5-year survival rate was 73.97%. Patients' clinic-pathologic characteristics are listed in Table 1. In general, 2 cohorts were well balanced with regard to base line characteristics. This study was approved by the Ethical Committee of the Fourth Hospital of Hebei Medical University.

### 2.2. Immunohistochemistry (IHC)

Immunohistochemical (IHC) staining was performed according to the standard protocol. CRC tumor tissues were embedded in paraffin blocks and cut into 4 *μ*m thick tissue sections. Xylene and a gradient of ethanol were used for dewaxing and rehydration. Endogenous peroxidase activity was eliminated and blocked using 3% H_2_O_2_ for 15 min and processed for antigen retrieval by high pressure cooking in an EDTA antigen retrieval solution (pH = 8.0) for 10 min. Subsequently, rabbit monoclonal antibodies against PD-1 (1 : 100, Abcam, Cambridge, USA) and Tim-3 (1 : 150, Abcam, Cambridge, USA) were added to the sections overnight at 4°C. Then, samples were washed with PBS and incubated with a rabbit anti-mouse secondary antibody (ZSGB-BIO, Beijing, China) at room temperature for 60 min. After further washing with PBS, samples were DAB stained (ZSGB-BIO, Beijing, China) at room temperature for 10 min. After dehydrating and drying, the sections were mounted with neutral gum and visualized on an inverted microscope (Olympus, Tokyo, Japan). In the course of the experiment, PBS was used as the negative control.

### 2.3. Result Determination of the Immunohistochemistry

Three high-power fields (200x) were randomly selected for each sample. Staining intensity was not accounted for, as only minor variations were observed. Specimens with stained cells ≥ 10% were considered high staining, and those with stained cells < 10% were considered low staining [22]. Extent of staining was scored as 1 (<33%), 2 (33%–66%), and 3 (>66%) according to the percentages of positive staining cells in relation to the carcinoma area. The staining intensity was scored as 1 (negative/weak), 2 (medium), or 3 (strong). The staining intensity was then multiplied by a multiple of the cell-positive percentage to generate an immunohistochemical score for each case. Samples having a final staining score of ≤3 were considered to be low and otherwise considered to be high [23]. The stained tissue sections were scored separately by two pathologists who were blinded to the clinic-pathological parameters.

### 2.4. Biological Interaction Network and Functional and Pathway Enrichment Analysis

Interactive relationships between PD-1 and Tim-3 were retrieved through the online STRING database (https://string-db.org/), which was used to study the interactions between proteins. Interaction of protein with a combined score > 0.700 was defined as statistically significant. Cytoscape (version 3.7.1) was applied to visualize PPI (protein-protein interaction) networks of interaction proteins. We then performed GO (Gene Ontology) and KEGG (Kyoto Encyclopedia of Genes and Genomes) pathway enrichment analysis of the interaction genes by using DAVID (Database for Annotation, Visualization, and Integrated Discovery, https://david.ncifcrf.gov/home.jsp; version 6.8) software, which consisted of comprehensive biological function annotation information. GO consists of three parts: the cellular component (CC), biological process (BP), and molecular function (MF).

### 2.5. Statistical Analysis

Statistical evaluation was conducted with SPSS statistical 21.0 software. The optimal cut-off values for the PD-1 and Tim-3 expressions in TCGA cohort were determined by ROC analysis. The chi-square test or Fisher exact test was used to analyze the relationship between clinic-pathological parameters and PD-1 and Tim-3 expressions. Survival curves were performed using the Kaplan-Meier method, and the univariate survival difference was determined by the logrank test. All significant factors from the univariate analysis were calculated in the multivariate analysis with a Cox regression model to identify independent survival factors. *P* < 0.05 was considered statistically significant.

## 3. Results

### 3.1. Clinical Characteristics of Patients with CRC in TCGA and FHHMU Cohorts

In TCGA cohort, the median age of all 378 CRC patients was 67 years old (ranging from 31 to 90 years). Only 112 patients (29.63%) were 60 years old or younger, and 266 patients (70.37%) were 61 and older. 194(51.32%) were male patients and 184(48.68%) were female patients.

In the FHHMU cohort, 35 (47.9%) patients were over 60 years old among the 73 patients. 36 (49.3%) were male patients and 37 (50.7%) were female patients. Tumor primary site, TNM stage, and histological type in TCGA and FHHMU cohorts are shown in Tables 1 and 2, respectively.

### 3.2. Expression Pattern of PD-1 and Tim-3 in the FHHMU Cohort

**Figure 1:**
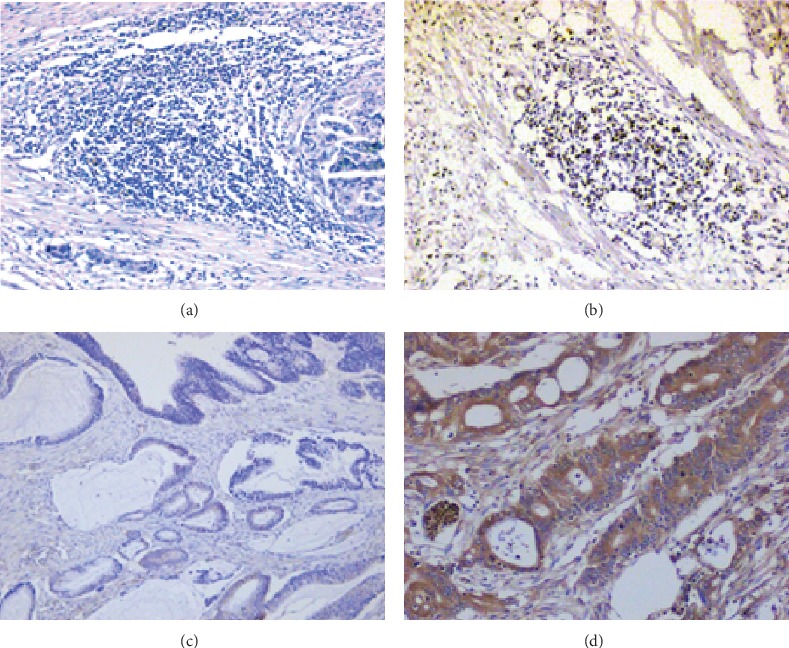
IHC staining of PD-1 and Tim-3 expressions in the FHHMU cohort (200x): (a) PD-1 low; (b) PD-1 high; (c) Tim-3 low; (d) Tim-3 high.

In the FHHMU cohort, the IHC staining was used to detect the expressions of PD-1 and Tim-3 in stage I-III CRC tumor tissue. PD-1 was expressed in a membrane-accentuated expression, and Tim-3 was expressed in the membrane and cytoplasm (Figure 1). The high expression rate of PD-1 and Tim-3 in stage I-III CRC tissue was 42.47% and 84.93%, respectively. High expression of PD-1 was observed in 31 patients (42.47%). Low expression of PD-1 was found in 42 cases (57.53%). 62 patients (84.93%) displayed a high expression of Tim-3, and the others showed a low expression of Tim-3.

### 3.3. Relationship between Clinic-Pathological Parameters and PD-1 or Tim-3 Expressions

In the cohort of TCGA, PD-1 expression was associated with the primary site and age (*P* < 0.05), whereas the Tim-3 expression was only correlated with the primary site (*P* < 0.05, Table 2). Higher expressions of PD-1 and Tim-3 were found in the right hemicolon tumor than the left hemicolon and rectum tumors. And the patients more than 60 years old had a higher expression of PD-1 than the others. In addition, there is no information whether these patients received neoadjuvant therapy in TCGA cohort.

In the FHHMU cohort, the PD-1 or Tim-3 expression was not associated with the primary site, age, and neoadjuvant treatment by a chi-square test or Fisher exact test. However, we found that the PD-1 expression was correlated with lymphatic metastasis and TNM (*P* < 0.05, Table 1). The expression of PD-1 was significantly higher in the patients with positive lymph node metastasis than in patients with negative lymph node metastasis (68.75% vs. 22.05%, *P* < 0.001). And the patients in clinical stage III had a higher PD-1 expression than those of stages I and II (68.75% vs. 8.33% or 27.59%, respectively, *P* < 0.001).

### 3.4. Coexpression of PD-1 and Tim-3

**Figure 2:**
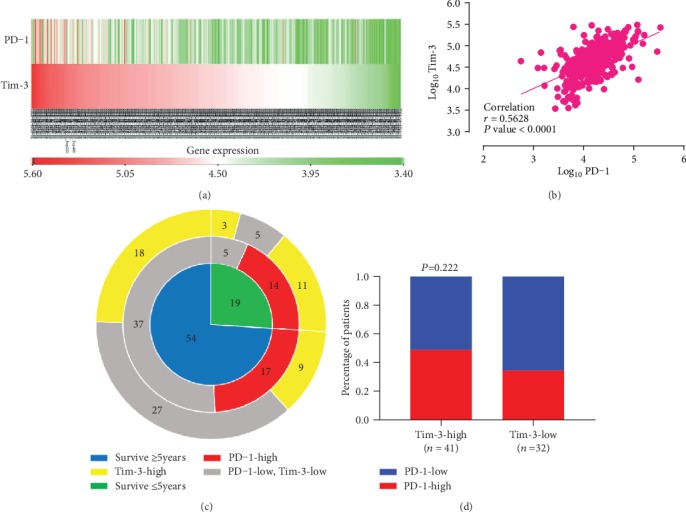
The relationships between PD-1 and Tim-3 expressions. (a, b) Expressions of PD-1 and Tim-3 and their correlation in TCGA cohort. (c, d) Expressions of PD-1 and Tim-3 and their correlation in the FHHMU cohort.

In TCGA cohort, we assessed the relationship between PD-1 and Tim-3 in mRNA levels and investigated the PD-1 and Tim-3 coexpression in CRC. Correlation analysis showed that PD-1 and Tim-3 expressions were positively correlated (*r* = 0.5682, *P* < 0.001, Figures 2(a) and 2(b)).

In the FHHMU cohort, the high expression of PD-1 with high and low levels of Tim-3 was 48.78% and 34.38%, respectively. A tendency of a positive correlation relationship between PD-1 and Tim-3 existed without significant statistical difference (*P* > 0.05, Figures 2(c) and 2(d)). A small sample size and the differences between gene and protein expressions may account for the inconsistent result between the above two cohorts.

### 3.5. Functional and Pathway Enrichment Analysis of PD-1 and Tim-3

**Figure 3:**
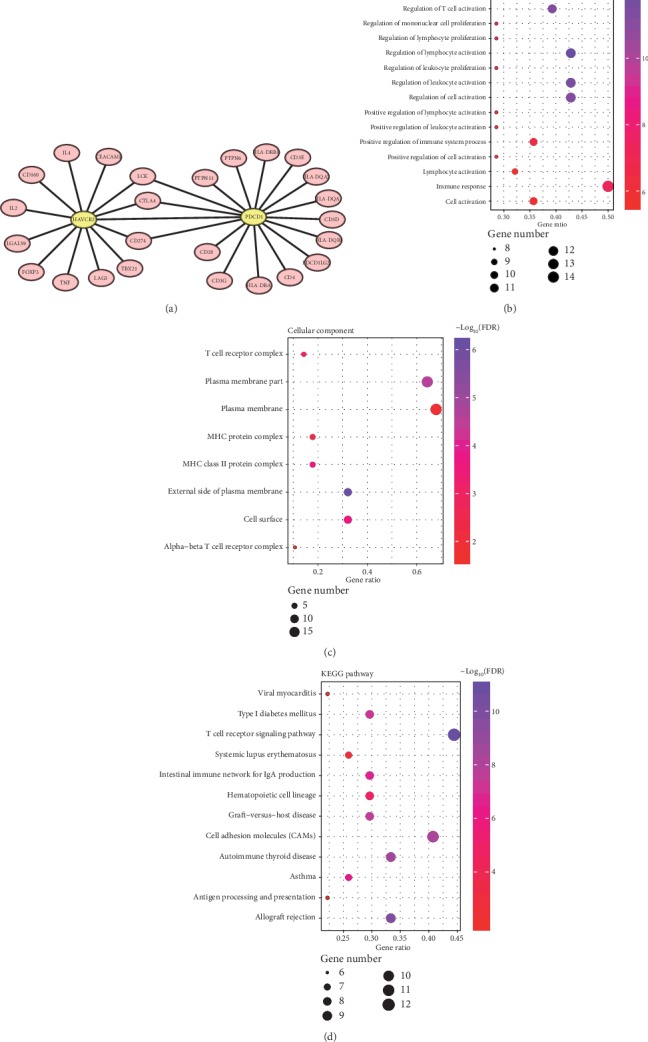
Functional and pathway enrichment analysis of PD-1 and Tim-3. (a) Visual summary of PD-1 and Tim-3 and biological interaction network (combined score ≥ 0.7); (b–d) the bubble diagrams display the enrichment results of the interaction genes of PD-1 and Tim-3.

To determine the biological interaction network of PD-1 and Tim-3, we used the tab network in STRING to show the interaction of PD-1 and Tim-3 proteins. The proteins were selected based on a combined score ≥ 0.7 in the STRING analysis (Figure 3(a)). Functional and pathway enrichment analysis was performed by using DAVID. Gene counts > 2 and FDR < 0.05 were set as the threshold. The results of the GO analysis showed that PD-1 and Tim-3 were mainly enriched in regulation of immune cell activation and proliferation of BP (Figure 3(b)), external side of the plasma membrane, plasma membrane part, cell surface, MHC class II protein complex, T cell receptor complex, MHC protein complex, plasma membrane, and *α*/*β* T cell receptor complex of CC (Figure 3(c)). Similarly, KEGG pathway analysis showed that PD-1 and Tim-3 participated primarily in the regulation of immune cell activation and proliferation, immune cell receptor complex, cell adhesion molecules, and T cell receptor signaling pathway (Figure 3(d)). With regard to the MF, we found that PD-1 and Tim-3 were only significantly enriched in MHC class II receptor activity. This suggested that the expression levels of PD-1 and Tim-3 were correlated with postoperative tumor recurrence, distant metastasis, and cancer progression.

### 3.6. PD-1 and Tim-3 Expressions Have a Negative Correspondence with the Prognosis of Stage I-III CRC

**Figure 4:**
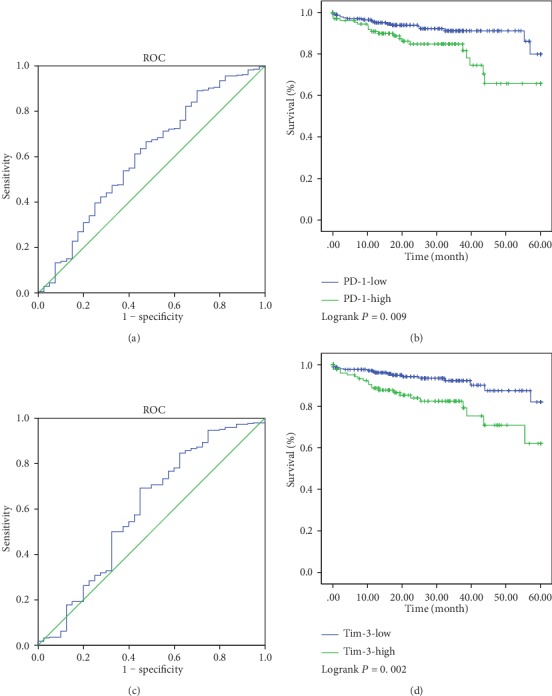
Determination of cut-off values of PD-1 and Tim-3 expressions in TCGA database and survival analyses. The optimal cut-off value for the PD-1 and Tim-3 expressions was determined by ROC analysis of 5-year OS using patients' data in TCGA database (a, c). The Kaplan-Meier plotters were used to analyze the 5-year OS (b, d). The optimal cut-off value for PD-1 was 4.33 (*P* = 0.009). The optimal cut-off value for Tim-3 was 4.80 (*P* = 0.002).

In the cohort of TCGA, patients were divided into 2 groups for further analysis (PD − 1 ≤ 4.33 and >4.33, Tim − 3 ≤ 4.80 and >4.80). ROC was used to identify the optimal cut-off values for the PD-1 and Tim-3 expressions in CRC tissue. The optimal cut-off values were 4.33 and 4.80, respectively (Figure 4). In TCGA cohort, PD-1 and Tim-3 mRNA expression levels were associated with the prognosis of CRC patients in terms of 5-year survival (*P* < 0.05, Figures 4(b) and 4(d)). Patients with a higher expression of PD-1 or Tim-3 had a significantly poorer prognosis than patients with lower expression.

**Figure 5:**
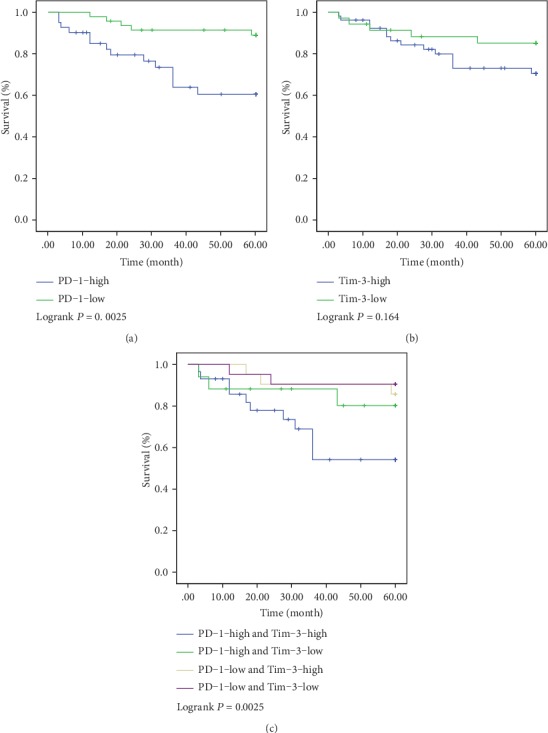
Kaplan-Meier analyses for the PD-1/Tim-3 expression and their correlation with clinical outcome in the FHHMU cohort. (a) 5 years of survival according to PD-1; (b) 5 years of survival according to Tim-3; (c) 5 years of survival according to PD-1 and Tim-3.

In the FHHMU cohort, a higher expression of PD-1 was related to the poor prognosis (5-year OS: 54.84% vs. 88.10%, *P* = 0.003, Figure 5(a)). The 5-year survival rate of patients with high expression of Tim-3 was 65.85%, while those patients with a low expression of Tim-3 of 84.38% showed no difference in statistics in terms of the 5-year survival rate (*P* > 0.05, Figure 5(b)). Comparing PD-1-high and Tim-3-high, PD-1-high and Tim-3-low, PD-1-low and Tim-3-high, and PD-1-low and Tim-3-low, the 5-year survival rates were 45.00%, 72.73%, 85.71%, and 90.48%, respectively. Notably, patients with both a high expression of PD-1 and high Tim-3 in CRC tumor tissues had the worst prognosis.

In addition, we also performed Cox regression to determine if PD-1 and/or Tim-3 expression were independent prognosticators in CRC (Table 3). In TCGA cohort and the FHHMU cohort, the univariate Cox regression model revealed that T stages, clinical stage, PD-1 expression, and Tim-3 expression were associated with the prognosis of CRC patients in terms of OS (*P* < 0.05). Multivariate analysis after adjustment indicated that only T stages, PD-1 expression, and Tim-3 expression were independent prognostic factors for OS in CRC patients (*P* < 0.05), and the clinical stage lost its significance (*P* > 0.05).

## 4. Discussion

Increasing studies have continuously confirmed the important role of the tumor immune microenvironment in the prognosis of tumor patients [24]. Immunotherapy has become the centre stage in the field of second-line treatment of cancer treatment, and anti-PD-1 therapy has shown objective responses in a variety of human malignancies, including lung cancer, melanoma, and bladder cancer [25, 26]. The efficacy of immunotherapy is closely related to the expression of PD-1 and Tim-3 in TILs of the tumor immune microenvironment [10, 27]. In this study, we analyzed the expressions of PD-1 and Tim-3 in stage I-III CRC patients treated with surgery and their clinical significance by TCGA database and the Fourth Hospital of Hebei Medical University (Hebei, China) cohort of patients. In TCGA database, we found that PD-1 was significantly associated with age, primary site, lymphatic metastasis, and poor prognosis. Tim-3 was significantly associated with the primary site and poor prognosis. In the enrolled patients, we found that the patients whose tumors had both high PD-1 and high Tim-3 expressions had the worst prognosis than the single high or double low expressions, which means coexpression of PD-1 and Tim-3 could be considered as predictive factors for prognosis in stage I-III CRC after curative resection.

However, the two cohorts were inconsistent in the relationship between clinic-pathological parameters and PD-1 or Tim-3 expressions. (1) The patients more than 60 years old had a higher level of PD-1 expression than the patients less than 60 years old in TCGA cohort. The small sample size in the FHHMU cohort may account for the result of no correlation between PD-1 and age. (2) There was a higher expression level of PD-1 and Tim-3 in the right hemicolon tumor than the left hemicolon and rectum tumors in TCGA cohort, which was different from the FHHMU cohort. (3) In the FHHMU cohort, PD-1 expression was associated with lymphatic metastasis, but the correlation could not be found in TCGA cohort.

Previous evidence suggests that the left hemicolon tumor and right hemicolon tumor may represent different pathological, genetic, and epidemiological characteristics [28]. For example, the right hemicolon tumor is generally poorly differentiated, displaying different molecular patterns, higher BRAF mutation, and MSI-high phenotype than the left hemicolon tumor [29, 30]. Yet, few people pay attention to the difference between the PD-1 and Tim-3 expressions in the left and right hemicolon tumors. Here, we found that the right-sided tumor had a higher expression level of PD-1 than the left-sided tumor in TCGA cohort. This may be an important reason for the poorer prognosis of the right hemicolon tumor than the left hemicolon tumor [31]. Because of the relatively small sample size, we did not detect the relationship between PD-1/Tim-3 expression and the primary site in the FHHMU cohort.

It is well known that lymphatic metastasis is an independent risk factor that affects the prognosis of CRC patients [31]. Lymphatic metastasis involves tumor cell transport in both lymphatic and blood vessels [29, 30]. When the immune system is fatigued, tumor cells highjack immune trafficking machinery to facilitate their own entry and transport within the lymphatic system; more importantly, immune cells lose the ability to kill tumor cells [32]. Herein, we found that PD-1 expression was associated with lymphatic metastasis in the FHHMU cohort. Emerging evidence suggests patients with a high expression of PD-1 tend to be more prone to lymph node metastasis [33]. However, we did not find the correlation between PD-1 and lymph node metastasis in TCGA cohort. We believed that the inconsistency between the two cohorts was likely due to two main reasons. Firstly, the PD-1 or Tim-3 mRNA level in TCGA database was detected by RNA sequencing using the whole RNA extracted from tumor tissue. Interestingly, we found the protein expressions of PD-1 and TIM-3 in the FHHMU cohort [5, 15]. Secondly, the translation of genes into proteins is also influenced by many factors, such as ubiquitination and acetylation [34].

Currently, PD-1 is regarded as a negative regulator of antitumor immunity [35]. It is reported that upregulation of PD-1 creates an immunosuppressive tumor microenvironment and helps cancer cells escape immune-mediated destruction [5]. A negative correlation between PD-1 expression and survival has been reported in non-small cell lung cancer, renal cell carcinoma, osteosarcoma, and breast cancer [36–38]. In this study, we evaluated the prognostic impact of PD-1 in stage I-III CRC tissue and confirmed that the high expression of PD-1 was associated with the poorer prognosis, which was similar to this study [39–41]. In addition, Miyamoto et al. found that patients with a high expression of PD-1 were more prone to lung metastasis in CRC [42].

In previous studies, researchers have found that high levels of Tim-3 indicate an exhausted T cell status and play an important role in the immune escape of malignant tumor cells [21, 35]. Prior investigations have also revealed that the infiltration with Tim-3^+^ exhausted TILs and ICOS^+^ Treg identifies the patients with poor prognosis in localized clear cell renal cell carcinoma [43]. The Tim-3/galectin-9 signaling pathway mediates T cell dysfunction and predicts poor prognosis in patients with hepatitis B virus-associated hepatocellular carcinoma [44]. However, unlike PD-1, we found that the expression of Tim-3 was not associated with patient prognosis in the FHHMU cohort. In TCGA data, we found that Tim-3 expression was significantly negatively correlated with the survival of stage I-III CRC patients. In addition, previous studies have shown that patients with a higher Tim-3 expression have a significant shorter survival time than those patients with a lower Tim-3 expression [19].

However, we discovered that the level of PD-1 had obviously positive correlation with the Tim-3 expression. Furthermore, the 5-year survival rate of CRC patients who had both high PD-1 and high Tim-3 expressions was 45.00%, which was significantly lower than those patients with PD-1-high and Tim-3-low, PD-1-low and Tim-3-high, and PD-1-low and Tim-3-low (72.73%, 85.71%, and 90.48%, respectively). Evidence confirmed that patients with coexpression of PD-1 and PD-L1 have a significantly worse recurrence-free survival, and coexpression of Tim-3 and carcinoembryonic antigen cell adhesion molecule 1 (CEACAM1) can result in the poor prognosis by promoting T cell exhaustion [39, 45]. Baitsch et al. found that upregulation of Tim-3 and PD-1 was associated with tumor antigen-specific CD8^+^ T cell dysfunction in melanoma patients [46]. Early trials of anti-PD1 antibodies in metastatic renal cell carcinoma patients have reported a 30% overall response rate and 20–25% prolonged response rate [47]. In addition, Tim-3 is associated with immunotherapy of metastatic renal cell carcinoma [48]. PD-1 can inhibit the proliferation of T cells and the production of related cytokines by the PI3K/AKT signaling pathway through immune cell fatigue [49]. When the pathways of PD-1/PD-L1 are activated, cancer cells could evade the immune response and continue to proliferate [50]. Nevertheless, the function of Tim-3 has not yet been demonstrated explicitly. Some researchers speculate that Tim-3 may directly facilitate tumor growth through the IL-6-STAT3 pathway *in vitro*[18, 51].

Herein, we found that PD-1, Tim-3, and the interaction between them played a role in immune regulation according to the functional and pathway enrichment analysis. Our results suggested that the functional network of PD-1 and Tim-3 primarily participated in the regulation of immune cell activation and proliferation, immune cell receptor complex, cell adhesion molecules, and T cell receptor signaling pathway. It is well known that regulation of immune cell activation and proliferation and the T cell receptor signaling pathway can significantly alter the immune status of the local microenvironment of the tumor [52]. Previous studies also suggest that high levels of PD-1 and Tim-3 in TILs indicate an exhausted T cell status [46, 53–55], which is consistent with the fact that PD-1 and Tim-3 participate in the immune escape of malignant tumor cells and cancer progression.

Of course, the present study had several limitations. Firstly, it was a single center and small sample size study, which may have an effect on the wider clinical applications. Secondly, an in-depth study of the underlying mechanisms for the role of PD-1 and Tim-3 in the prognosis of CRC should be conducted. Animal model or cell experiments are further needed to investigate the deeper molecular mechanism of PD-1 and Tim-3 in the CRC diagnosis and assess the prognostic significance of PD-1 and Tim-3. Thirdly, only immunohistochemistry was used to detect the expressions of PD-1 and Tim-3 in CRC. Fourthly, flow cytometry is further needed to identify the cell location (such as TILs and TLs) of PD-1 and Tim-3 expressions in the stage I-III CRC tissue. Fifthly, we analyzed the expressions of PD-1 and Tim-3 using mRNA levels vs. protein expression in immunohistochemistry. Generally, the biological function of the gene and protein is equivalent, besides expression quantity. In addition, the protein expression analysis is common in clinical practice. Therefore, further analysis of PD-1 and Tim-3 using protein levels in TCGA dataset vs. protein expression in immunohistochemistry or protein western blot is needed.

## 5. Conclusion

In summary, high PD-1 and Tim-3 expressions in stage I-III CRC tumor may be associated with poor prognosis. Patients with coexpression of PD-1 and Tim-3 may have a significantly worse prognosis. The main reason may be that T cell fatigue leads to the metastasis of tumor cells in lymph nodes, but its exact mechanism needs further investigation.

## Figures and Tables

**Table 1:** Comparison of baseline clinic-pathological characteristics based on PD-1 and Tim-3 expressions of CRC patients in the FHHMU cohort.

	Cases (no.(%))	PD-1			Tim-3		
		High (no.(%))	*χ* ^2^ (Fisher)	*P*	High (no.(%))	*χ* ^2^ (Fisher)	*P*
Age (years)			2.211	0.161		0.096	0.816
≤60	38(52.05)	13(34.21)			22(57.89)		
>60	35(47.95)	18(51.43)			19(54.29)		
Gender			0.632	0.668		0.011	1.000
Male	36(49.32)	16(44.44)			20(55.56)		
Female	37(50.68)	15(40.54)			21(56.76)		
Primary site			0.608	0.738		0.408	0.816
Right hemicolon	23(31.50)	11(47.83)			12(52.17)		
Left hemicolon	22(30.14)	8(36.36)			12(54.55)		
Rectum	28(38.36)	12(42.86)			17(60.71)		
T stage			3.245	0.355		3.117	0.374
T1	2(2.74)	1(50.00)			0		
T2	14(19.18)	3(21.43)			8(57.14)		
T3	22(30.14)	11(50.00)			14(63.64)		
T4	35(47.95)	16(45.71)			19(54.29)		
N stage			16.111	<0.001		0.214	0.812
N0	41(56.16)	9(22.05)			24(58.54)		
N+	32(43.84)	22(68.75)			17(53.13)		
Clinical stage			17.398	<0.001		0.716	0.699
I	12(16.44)	1(8.33)			6(50.00)		
II	29(39.73)	8(27.59)			18(62.07)		
III	32(43.84)	22(68.75)			17(53.13)		
Neoadjuvant therapy			4.995	0.082		0.149	0.928
Yes	19(26.03)	11(57.89)			11(57.89)		
No	46(63.01)	15(32.61)			26(56.52)		
Unknown	8(10.96)	5(62.50)			4(50.00)		
Total	73(100)	31(42.47)			41(56.16)		

**Table 2:** Comparison of baseline clinic-pathological characteristics based on PD-1 and Tim-3 expressions of CRC patients in TCGA cohort.

	Cases (no.(%))	PD-1			Tim-3		
		High (no.(%))	*χ* ^2^ (Fisher)	*P*	High (no.(%))	*χ* ^2^ (Fisher)	*P*
Age(years)			10.413	<0.001		0.102	0.812
≤60	112(29.63)	26(23.21)			36(32.14)		
>60	266(70.37)	108(40.60)			90(33.83)		
Gender			0.632	0.668		1.530	0.231
Male	194(51.32)	71(36.60)			59(30.41)		
Female	184(48.68)	63(34.24)			67(36.41)		
Primary site			11.410	0.003		11.354	0.003
Right hemicolon	162(42.86)	71(43.83)			69(43.59)		
Left hemicolon	109(28.83)	26(23.85)			31(28.44)		
Rectum	107(28.31)	37(34.60)			26(24.30)		
T stage			0.156	0.984		4.126	0.248
T1	13(3.44)	4(30.77)			2(15.38)		
T2	83(21.96)	29(34.94)			23(27.71)		
T3	260(68.78)	93(35.77)			92(35.38)		
T4	22(5.82)	8(36.36)			9(40.91)		
N stage			1.441	0.248		0.742	0.413
N0	259(68.52)	97(37.45)			90(34.75)		
N+	119(31.48)	37(31.09)			36(30.25)		
Clinical stage			1.454	0.483		4.815	0.090
I	87(23.02)	33(37.93)			23(26.44)		
II	172(45.50)	64(37.21)			67(39.41)		
III	119(31.48)	37(31.09)			36(30.25)		
Histological type			0.221	0.620		3.292	0.088
Adenocarcinoma	334(88.36)	117(35.03)			106(31.74)		
Mucinous	44(11.64)	17(38.64)			20(45.45)		
Total	378(100)	134(35.45)			126(33.33)		

**Table 3:** Univariate and multivariate Cox proportional hazards analysis of OS for patients with CRC in TCGA and FHHMU cohorts.

Variables	Univariate analysis			Multivariate analysis		
	HR	95% CI	*P*	HR	95% CI	*P*
Age(years)						
≤60	Reference		0.908			
>60	1.032	0.605-1.761				
Gender						
Male	Reference		0.108			
Female	0.654	0.390-1.097				
Primary site						
Right hemicolon	Reference		0.414			
Left hemicolon	1.509	0.719-3.167				
Rectum	1.109	0.493-2.497				
T stage						
T1	Reference		<0.001	Reference		0.010
T2	2.485	1.349-4.578		1.609	0.698-3.710	
T3	2.247	1.549-3.260		1.568	0.802-3.066	
T4	2.065	1.474-2.893		1.975	1.408-2.772	
N stage						
N0	Reference		0.427			
N+	1.233	0.735-2.069				
Clinical stage						
I	Reference		<0.001	Reference		0.588
II	1.407	1.067-1.855		1.530	0.737-2.472	
III	1.095	0.864-1.387		0.956	0.727-1.257	
PD-1						
Low	Reference	1.640-4.636	<0.001	Reference	1.085-3.244	0.024
High	2.757			1.877		
Tim-3						
Low	Reference		<0.001	Reference		0.008
High	2.580	1.524-4.366		1.402	1.212-3.676	

## Data Availability

The data of TCGA database was obtained from the website of Cancer Genomics Browser of University of California Santa Cruz (https://genome-cancer.ucsc.edu/).The data of the Fourth Hospital of Hebei Medical University (FHHMU) cohort used to support the findings of this study are available from the corresponding author upon request.

## Abbreviations

BP: Biological process
CEACAM1: Carcinoembryonic antigen cell adhesion molecule 1
CC: Cellular component
CRC: Colorectal cancer
DAVID: Database for annotation, visualization, and integrated discovery
FHHMU: Fourth Hospital of Hebei Medical University
GO: Gene Ontology
IHC: Immunohistochemistry
KEGG: Kyoto Encyclopedia of Genes and Genomes
MF: Molecular function
OS: Overall survival
PPI: Protein-protein interaction
PCNSL: Primary central nervous system lymphoma
PD-1: Programmed cell death receptor 1
Tim-3: T cell immunoglobulin mucin-3
TCGA: The Cancer Genome Atlas
TCs: Tumor cells
TILs: Tumor-infiltrating cells.
